# Comparison of Two Statistical Methods to Determine Normal Range of Androgen Hormones: K-Means Cluster Analysis and Receiver Operating Characteristic Curve 

**Published:** 2018-06

**Authors:** Fahimeh Ramezani-Tehrani, Khaled Rahmani, Ali Moradi, Seyed Ali Montazeri, Razieh Bidhendi-Yarandi, Fatemeh Darabi

**Affiliations:** 1 Reproductive Endocrinology Research Center, Research Institute for Endocrine Sciences, Shahid Beheshti University of Medical Sciences, Tehran, Iran; 2 Department of Community Medicine, Social Determinants of Health Research Center, Research Institute for Health Development, Kurdistan University of Medical Sciences, Sanandaj, Iran; 3 Health Deputy, Hamadan University of Medical Sciences, Hamadan, Iran; 4 Department of Public Health, Asadabad School of Medical Sciences, Asadabad, Iran

**Keywords:** Androgen Hormones, K-Means Cluster Analysis, Receiver Operating Characteristic Curve

## Abstract

**Objective:** To assess and compare the normal ranges of androgen hormones level, total testosterone (TT), free testosterone (FT), dehydrotestosterone (DHT), androstenedione (A4), dehydroepiandrosterone (DHEA), and dehydroepiandrosterone sulfate (DHEAS), in Iranian women based on different statistical methods.

**Materials and methods:** This study was conducted on previous data collected in Iranian PCOS Prevalence Study, which details have been published before. A total of 1772 women of 18-45 years were recruited from urban areas of five randomly selected provinces in different geographic regions of Iran. The natural range of androgen hormones was determined and compared by two statistical methods including k-means cluster analysis, and receiver operating characteristic curve.

**Results:** In women younger than 35 years old with any BMI, cut-off points obtained for FAI hormone were in lower percentiles; however, in older women, the results of the two methods were almost the same. Cut-off points of DHEAS in under 35 years old women of normal and obese weight and women older than 35 years old with normal weight calculated by ROC curve method was in higher percentiles than that in the cluster analysis method. In >35 years obese women, obtained cut-off points for DHEAS ROC curve was in lower percentiles in comparison to cluster analysis

**Conclusion:** Although our study depicts the differences among the cutoff values among two statistical methods; however, lacking a gold standard test to define hyperandrogenism, we need further studies to obtain more comprehensive results.

## Introduction

Hyperandrogenism (i.e. androgen excess), the most common endocrinopathy in reproductive women, refers to high level of plasma androgens produced in adrenal glands and ovaries (1). Hyperandrogenism strongly associates with polycystic ovary syndrome (PCOS), which risk of development in reproductive-age women is 7-12 %(2). The outcomes include infertility, endometrial cancer, delayed menopause, insulin resistance (IR), diabetes mellitus (DM), dyslipidemia, and cardiovascular disease (CVD) (3-7).

Diagnosis mainly depends on clinical presentations and laboratory measures. The most common clinical presentations include hirsutism, virilization, acne vulgaris, and androgenic alopecia (8). Almost 80-90% of women with hirsutism finally diagnosed with hyperandrogenism(9). Biochemical measures include total testosterone (TT), free testosterone (FT), dehydrotestosterone (DHT), androstenedione (A4), dehydroepiandrosterone (DHEA), and dehydroepiandrosterone sulfate (DHEAS) (1, 10).

The amount of abnormal hair growth in a woman’s body or face and the mean level of androgen hormones vary among different population and ethnicities(11, 12). Thus, it could be better to define normal ranges of these hormones special to each ethnicity and population.

Different methods are used to explore the normal range of androgen hormones (13). One of the commonest and simplest ways is to apply 5^th^ and 95^th^ percentiles of the hormonal level in the population as the lower and upper limits of normal ranges, respectively (14). However, it could result in overestimation of hyperandrogenism in a population like Iranian women, whose mean androgen levels are higher than that in other populations, and underestimation otherwise(15). Moreover, different diagnostic criteria, screening methods, recruitments of subjects, study designs, measurements, data collection and analyses, and interpretation of the results could contribute to various ranges of androgen hormone level used as normal in different populations (16-18)- as the prevalence of PCOS (as an important association of hyperandrogenism) varies from 2% to 26% in different populations (3, 16, 19).

On the basis of the aforementioned shortcomings, we have used data of a population-based study- Iranian PCOS Prevalence Study- to assess and compare the normal ranges of androgen levels in Iranian women based on different statistical methods: k-means cluster analysis, and receiver operating characteristics (ROC) curve.

## Materials and methods

This study was conducted on data collected in Iranian PCOS Prevalence Study, which details have been published before (15). In summary, 1772 women of 18-45 years were recruited from urban areas of five randomly selected provinces in different geographic regions of Iran. Following informed written consent, trained midwives completed a standard questionnaire, which include demographic features, socio-economic status, medical, surgical, and familial history, during face-to-face interviews under supervision of a single gynecologist. 

Exclusion criteria were as follows: menopause, pregnancy at the time of the evaluation (n = 59), hyperprolactinemia or thyroid diseases (n = 37), hirsutism (n = 347), PCOS by Rotterdam criteria (n = 223), and incomplete data (n = 173). The number of studied participants in previous study were 929 women that we excluded six cases due to lack of required information for the analysis, and 923women constituted our study participants. Those who had hirsutism scores 0-1 were in the Control group (n = 423), and those with 2-7 scores (n = 500) consisted the main group (20).

The ethical review board of the Research Institute for Endocrine Sciences approved the study proposal and design.

Blood pressure, anthropometric, hormonal and metabolic measurements were assessed for all study subjects at the interview day. All subjects underwent transvaginal scan or transabdominal ultrasonography of the ovaries using a 3.5-MHz transabdominal and 5-MHz transvaginal transducer, respectively. Ultrasound was performed at the same day as blood samples were collected, i.e. on the 2^nd^or 3^rd^day of her spontaneous or progesterone-induced menstruation.

Dehydroepiandrosterone sulfate (DHEAS) was measured by enzyme immunoassay (EIA, DRG Instruments, GmbH, Germany); 17-hydroxyprogesterone (17-OHP), TT and A4 were measured by EIA (Diagnostic biochem Canada Co. Ontario, Canada). Sex hormone binding globulin (SHBG) was measured by immunoenzymometric assay (IEMA, Diagnostic biochem Canada Co. Ontario, Canada). All ELISA tests were performed using the Sunrise ELISA reader (Tecan Co. Salzburg, Austria). The intra- and inter-assay coefficients of variation for TT were 1.7% and 2.3%, respectively, These values for DHEAS were 1.9 and 2.5%, for 17-OHP were 4.8 and 6.8%,for SHBG were 0.8 and 2.4%, and for A4 were 4.5 and 6.8%, respectively. Free androgen index (FAI) calculated using this formula:

$\mathrm{FAI}=100\times \frac{\mathrm{TT}(\mathrm{nmol}/l)}{\mathrm{SHBG}(\mathrm{nmol}/l)}$

All descriptive and analytical analyses were conducted using SPSS software (IBM Corp. Released 2011. IBM SPSS Statistics for Windows, Version 20.0. Armonk, NY: IBM Corp.)

In the first part of the analyses, mean (Standard Deviation) was recorded for continuous variables and frequency (percent) in each category for categorical variables. In the second, normal limit of androgen hormones was determined by comparing two methods: k-means cluster analysis, and Receiver Operating Characteristic (ROC) curve.

***K-means cluster analysis:*** Cluster analysis is a useful method to discriminate between groups especially when there is no gold standard of normality. Actually, by this method we make groups, i.e. clusters, which members are internally homogenous (within a cluster) but externally different (between clusters). To identify the normative cut-off values, the single minimum value of the higher cluster is chosen to be the best estimate (21).

***K-means cluster analysis algorithm:*** In order to define 3 ranges of androgen, we used K means clustering. Here to define clusters as low, moderate and high level (3 clusters) 3-means clustering algorithm was applied. First, we chose individual’s values of androgen as the initial seeds in a way that they had the furthest Euclidean distances of each other. Therefore, in the first stage these values are the centroid of the defined clusters. Second, each remaining individual would be compared with these centroids of the clusters according to Euclidean distance; the assignment criteria would be the closest distance. Each time by recruiting a new member the centroid of the clusters will be updated. Finally, to check whether individuals have been assigned to the right cluster, we compared each individual’s androgen value Euclidean distance to its own cluster centroid and the rest of clusters. It should have the closest distance to its own cluster, otherwise relocation should be considered. In this way, we clustered individuals in 3 ranges of androgen, which cluster centroid represented the mean of each level. These clusters are completely distinct of each other; however, they are internally homogenous. Then, minimum value for higher cluster shows the cut point.

***Receiving operating curve (ROC) analysis:*** ROC curve is used to discriminate between two groups when there is a binary outcome (22). It seems that in case of determining a cut-off value when there is no apparent outcome, this method is less efficient. By the way, to define the best cut-off value for hyperandrogenism hormones we used Hyperandrogenemia variable as the binary outcome. The point in which the ROC curve has maximum sensitivity and specificity was chosen as the best cut-off value. Youden index, which is defined as maximum value of sensitivity + specificity, was used to obtain cut-off point. In addition, we could find percentiles of the population.

Each method has its own preliminary assumption that can limit its application. Here we study the pros and cons of them and their convenience of applying. 

None of them require normality distribution assumption, but it may cause some problem when data is skewed and outliers exist.

Cluster analysis, which follows a very simple algorithm, started by initial seed chosen by expert ideas. Therefore, it could have different results for different subjective ideas. It is very sensitive to initial seeds to reduce this effect algorithm can be run, iteratively. It does not consider the probability distribution of the data; just a simple mathematical algorithm (according to Euclidean distance) was applied to cluster data; which is not reliable, especially when variation of the measures is low, discriminating clusters faced with problems. In addition, experts could not make any inference since its context is not probabilistic, and all indices proposed via this method are heuristics (23).

Despite data distribution, non-parametric approaches were applied in this method. Therefore, inferences could be made for indices estimated. But, sometimes drawing ROC curve face with some complexities, for instance it necessitates extrapolation when data are not normal, statistical method used for curve extrapolation affect AUC calculation as well (24).

## Results

Of total 923 women selected for this study, 423 women consist the Controlgroup (hirsutism score 0-1) and the remaining women with hirsutism score of 2-7 consist the main group.To test Normality of hormonal profile Kolmogorov-Smirnov and Shpiro-Wilk test were applied which found significant therefore Mann-Whitney U non-parametric test was used to test the differences. The characteristics of all study subjects are shown in Table 1.

K-means Cluster analysis**:**Mean, median, and percentiles of FAI, TT, DHEAS, and A4 decreased significantly in older women (p < 0.05). The level of these hormones differs significantly between two age groups (p < 0.05). 

Mean, median, 10^th^, and 95th percentiles of DHEAS and A4 had a negative relationship with BMI; i.e. these amounts decreased significantly in higher BMI levels. Although FAI cut-off points in normal-weight women were significantly different from those in obese ones regardless of age, no difference was seen in all androgen hormones (Table 2).

The cut-off points of FAI, TT, DHEAS, and A4 in total population resemblesthe 95th percentile; however, in the Totalbase population the cut-off points of these hormones were 79.3%, 74.1%, 96.7%, and 71%, respectively (Table 3).

**Table 1:** Characteristics of study subjects

**Variables**	**Total (n = 923)**	**Control group (n = 423)**	C **P _value**
Age; year	34.3 ± 7.3	35.3 ± 7.4	0.000^*^
Body mass index (BMI)	26.8 ± 5.0	26.7 ± 5.0	0.000^*^
Age of menarche; year	13.4 ± 1.5	13.3 ± 1.4	0.143
WC	85.0 ± 12.1	84.6 ± 11.9	0.000^*^
Parity	2.5 ± 1.4	2.5 ± 1.3	0.065
FPG	88.9 ± 26.0	88.4 ± 21.3	0.000^*^
Free androgen index (FAI)	3.6 ± 2.6	2.6 ± 1.4	0.000^*^
Age group < 35	3.0 ± 1.9	3.7 ± 1.7	0.000^*^
Age group >= 35	2.4 ± 1.5	3.1 ± 2.6	0.000^*^
BMI group: Normal	2.8 ± 1.9	3.4 ± 2.4	0.000^*^
BMI group: Obese	3.5 ± 1.6	3.4 ± 1.3	0.000^*^
BMI group: Over weight	2.1 ± 1.6	3.5 ± 2.8	0.000^*^
Dehydroepiandrosterone sulfate (DHEAS)	164.3 ± 105.1	138.2 ± 80.4	0.000^*^
Age group < 35	207.5 ± 124	191.5 ± 105	0.000^*^
Age group >= 35	137.2 ± 84	125.4 ± 78	0.000^*^
BMI group: Normal	201.7 ± 129	169.5 ± 92	0.000^*^
BMI group: Obese	170.3 ± 110	129.5 ± 101	0.000^*^
BMI group: Over weight	159.5 ± 89	160.2 ± 77	0.000^*^
Total testosterone (TT)	0.63 ± 0.6	0.5 ± 0.2	0.000^*^
Age group < 35	0.55 ± 0.43	0.65 ± 0.3	0.000^*^
Age group >= 35	0.44 ± 0.33	0.59 ± 0.9	0.000^*^
BMI group: Normal	0.56 ± 0.47	0.62 ± 0.5	0.000^*^
BMI group: Obese	0.49 ± 0.30	0.57 ± 0.4	0.000^*^
BMI group: Over weight	0.47 ± 0.39	0.66 ± 0.9	0.000^*^
Androstenedione (A4)	1.5 ± 0.06	1.2 ± 0.5	0.000^*^
Age group < 35	1.9 ± 0.66	1.68 ± 0.5	0.000^*^
Age group > = 35	1.5 ± 0.68	1.28 ± 0.7	0.000^*^
BMI group: Normal	1.9 ± 0.69	1.57 ± 0.6	0.000^*^
BMI group: Obese	1.6 ± 0.74	1.46 ± 0.7	0.000^*^
BMI group: Over weight	1.6 ± 0.63	1.34 ± 0.7	0.000^*^

C Obtained from Mann-Whitney U non-parametric test of two independent sample

**Table 2:** Comparison of cut-off points for FAI, TT, DHEAS and A4 Hormones across age and BMI groups with cluster analysis and ROC curve methods in total population

**Hormone**	**Method**		**Age < 35**			**Age >= 35**		
			**Normal**	**Overweight**	**Obese**	**Normal**	**Overweight**	**Obese**
FAI	Cluster Analysis	Cut point	5.10	4.85	6.31	4.48	4.86	4.79
		Percentile	81	76	81	74	82	78
	ROC Curve	Cut point (Youden Index)	2.91 (1.6)	2.48 (1.3)	2.96 (1.7)	4.76 (1.7)	5.18 (1.9)	5.09 (1.9)
		Percentile	63	56	63	83	87	87
TT	Cluster Analysis	Cut point	0.78	1	0.87	1.84	2.10	0.71
		Percentile	66	91	82	98	98	76
	ROC Curve	Cut point (Youden Index)	0.92 (1.2)	0.83 (1.5)	0.77 (1.5)	0.86 (1.6)	0.83 (1.6)	1.22 (1.3)
		Percentile	93	88	83	90	88	96
DHEAS	Cluster Analysis	Cut point	226.02	12.20	212.80	181.21	1.94	148.91
		Percentile	69	63	71	74	77	64
	ROC Curve	Cut point (Youden Index)	238.60 (1.3)	210.01 (1.4)	186.71 (1.5)	217.61 (1.6)	184.51 (1.5)	131.42 (1.3)
		Percentile	89	78	67	82	72	46
A_4_	Cluster Analysis	Cut point	2.31	1.98	2.08	1.61	2.01	1.90
		Percentile	78	64	77	69	87	83
	ROC Curve	Cut point (Youden Index)	2.11 (1.6)	2.00 (1.1)	2.41 (1.4)	2.31 (1.4)	2.00 (1.4)	2.00 (1.4)
		Percentile	79	74	90	87	74	74

***ROC curve results:*** Charts 1 to 5 show the area under ROC curve for FAI, TT, DHEAS, and A4 in studied women. These charts are drawn based on different percentiles (50^th^, 55^th^, 60^th^, 70^th^, and 85^th^. ROC curve results showed that cut-off points of FAI, TT, and DHEAS in women with any BMI (natural, overweight, or obese) were statistically different in two age groups. 

**Table 3:** Compare cut points of FAI, TT, DHEAS and A4 Hormones across BMI groups with cluster analysis, and ROC curve in total population and Control group

**Hormone**	**Method**		**Total Population** **N=923**			**Control Group** **N=423**		
			**Normal**	**Overweight**	**Obese**	**Normal**	**Overweight**	**Obese**
FAI	Cluster Analysis	Cut point	4.90	3.96	3.84	3.94	4.17	4.84
		Percentile	80	76	75	79.3	83	94
	ROC Curve	Cut point (Youden Index)	3.96 (1.6)	3.84 (1.7)	3.78 (1.6)	3.51 (1.7)	5.45 (1.6)	5.36 (1.8)
		Percentile	76	75	73	81	96	96
TT	Cluster Analysis	Cut point	1.60	1.70	0.76	0.66	1.83	2.01
		Percentile	66	76	77	74.1	79	98
	ROC Curve	Cut point (Youden Index)	0.98 (1.3)	0.88 (1.5)	0.82 (1.7)	0.65 (1.6)	0.73 (1.7)	1.11 (1.3)
		Percentile	95	91	89	80	89	99
DHEAS	Cluster Analysis	Cut point	214.80	198.60	195.31	275.21	245.65	207.31
		Percentile	84	67	74	97	89	79
	ROC Curve	Cut point (Youden Index)	236.03 (1.2)	227.98 (1.5)	205.10 (1.5)	218.32 (1.4)	203.12 (1.4)	145.23 (1.5)
		Percentile	85	86	80	87	83	68
A_4_	Cluster Analysis	Cut point	2.11	1.87	1.94	1.53	1.91	1.98
		Percentile	73	72	84	71	82	89
	ROC Curve	Cut point (Youden Index)	2.23 (1.6)	1.91 (1.5)	1.89 (1.6)	2.11 (1.5)	1.92 (1.3)	1.71 (1.4)
		Percentile	73	72	72	78	81	79

Cut-off points of A4 in overweight and normal BMI women were similar in two age groups. Hyperandogenemia considered as the State variable (Table 2). 

***Comparison of two methods (cluster analysis and ROC curve):***In women younger than 35 years old with any BMI, cut-off points obtained for FAI hormone were in lower percentiles; however, in older women, the results of the three methods were almost the same.

Cut-off points of DHEAS in under 35 years old women of normal and obese weight and women older than 35 years old with normal weight calculated by ROC curve methods was in higher percentiles than that in the cluster analysis method. In > 35 years obese women, obtained cut-off points for DHEAS using ROC curve was in lower percentiles in comparison to cluster analysis. 

Obese women have higher cut-off points of FAI hormone in base group according to results of the two methods results. Obese women have a higher cut-off point in two population groups. Cut-off points of DHEAS in women with normal weight using three methods were in higher percentiles. Obtained cut-off points using two different methods had not specific trend.

## Discussion

In this study we compared results of two methods: cluster analysis and ROC curve for assessing the normal limits of androgen hormones. The normal limits of FAI were 4.90 (80^th^ percentile), and 3.96 (76^th^ percentile) according to cluster analysis, and ROC curve, respectively. The cutoff of TT was as follows: 1.60 (66^th^ percentile), and 0.98 (95^th^ percentile) based on cluster analysis, and ROC curve, respectively. The cut-off points of DHEAS were 214.80 (84^th^), and 236.03 (85^th^) derived from cluster analysis, and ROC curve, respectively. According to cluster analysis, and ROC curve, the normal limits of A4 were 2.11 (73^rd^ percentile), and 2.23 (73^rd^ percentile), respectively.

Cluster analysis has a complementary perspective, which underlying logic is classification. In cluster analysis, this means that research units (individuals or groups), which are located in the rows of the data matrix, are classified in clusters. Variables are homogenous in each cluster and distinct from those in other clusters.

Despite K-means cluster analysis is one of the most common methods used in cluster analyses, the end result might not differ from other methods in cluster analysis. K-mean cluster analysis might suffer from some shortcomings: First, the final results depend on the choice of initial clusters. Second, there are no certain processes to initial computation cluster centers. Third, if the number of data in one cluster is zero in replication of algorithms, there is no way to change and continue this way. Finally, the number of clusters is not always clear in the beginning of studies; however, we did not have such a problem in this study (25).

Many studies have used these methods to determine normal limits of various variables. Sharifi et al. used the ROC curve to detect vitamin D deficiency based on homeostatic model assessment-insulin resistance (HOMA-IR) (26). In the study of Burns et al. ROC curves were used to determine pedometer step count cut-points that associated with at least 30 min of MVPA during school hours (27). Zhao et al. used a cluster analysis to define what is abnormal without the use of potentially biased and controversial definitions of hirsutism (28).

One of the major limitations of this study was lack of a gold standard method to detect hyperandrogenism to compare the applicability of mentioned statistical methods for diagnosing hyperandrogenism. However, we tried to access representative samples by subject recruitment from provinces located in different area of the country; so our results could help clinicians and epidemiologist to compare these statistical methods to detect hyperandrogenism.

In conclusion, the normal limits of androgen hormones are determined using two statistical analyses: cluster analysis and ROC curve. The exact cut-off of androgen levels to determine hyperandrogenism depends on the statistical analysis used for that purpose. Our study depicts the differences among the cutoff values among these statistical methods; however, lacking a gold standard test to define hyperandrogenism, necessitate further studies in this field to obtain more comprehensive results.

## Conclusion

Our data depicts the differences among the cutoff values among two statistical methods, cluster analysis and ROC curve, to determine the normal limits of androgen hormones. Based on the results, the exact cut-off of androgen levels to determine hyperandrogenism depends on the statistical analysis used for that purpose.; however, lacking a gold standard test to define hyperandrogenism, we need further studies to obtain more comprehensive results.
